# Drug-Related Problems among Hospitalized Surgical Elderly Patients in China

**DOI:** 10.1155/2021/8830606

**Published:** 2021-02-15

**Authors:** Long Meng, Can Qu, Xia Qin, Huali Huang, Yongsheng Hu, Feng Qiu, Shusen Sun

**Affiliations:** ^1^Department of Pharmacy, The First Affiliated Hospital of Chongqing Medical University, Chongqing, China; ^2^Department of Pharmacy Practice, College of Pharmacy and Health Sciences, Western New England University, Springfield, USA; ^3^Department of Pharmacy, Xiangya Hospital, Central South University, Changsha, Hunan, China; ^4^The Hunan Institute of Pharmacy Practice and Clinical Research, Changsha, Hunan, China

## Abstract

There is a lack of data on drug-related problems (DRPs) among elderly patients from surgical departments. The current study is aimed at identifying and categorizing types of DRPs and assessing the severities of the DRPs. Medication orders for hospitalized patients aged ≥65 years from six surgery departments were reviewed to determine DRPs over 6 months in a tertiary teaching hospital of Chongqing, China. DRPs were classified based on the Pharmaceutical Care Network Europe classification V8.02. The severity ratings of the DRPs were assessed using the National Coordinating Council for Medication Error Reporting and Prevention classification. A total of 53,231 medication orders from 1,707 elderly patients were reviewed, and 1,061 DRPs were identified. Treatment safety (44.9%) was the most common DRP type. Drug selection (43.1%) and dose selection (43.1%) were the major causes of DRPs. A total of 75.1% of the DRPs were classified into severity categories B to D (causing no or potential harm), and 24.9% were classified as categories E to H (causing actual harm). DRPs are common in hospitalized elderly surgical patients. Pharmacists should provide medication order reviews in this vulnerable patient population.

## 1. Introduction

A drug-related problem (DRP) is defined as “an event or circumstance involving drug therapy that actually or potentially interferes with desired health outcomes” [1]. Elderly patients often take multiple medications, and they are more likely to experience DRPs, which may increase the risk of morbidity and mortality [2]. The Pharmaceutical Care Network Europe (PCNE) DRP classification can categorize DRPs in various settings.

Since 2018, the PCNE DRP classification has been introduced into clinical pharmacy services at Chinese hospitals, and it has gained popularity as a DRP documentation tool. Studies have demonstrated that pharmacist-provided medication review can detect and resolve DRPs in diverse patient populations at Chinese hospitals, such as surgical patients [3], elderly patients [4], stroke patients [5], and patients with respiratory diseases [6, 7]. However, there has been no analysis of assessing DRPs in hospitalized elderly surgical patients from China. We established a medication review service at our hospital to detect and resolve DRPs in hospitalized patients. Our previous published study (including 10,643 patients) indicates that the medication review service is essential to detect DRPs in surgical patients and prevent harm to these DRPs [3]. This report is a subgroup analysis of the DRPs presented in elderly patients from this published study.

## 2. Aim of the Study

The primary aim was to identify and categorize DRPs (types, causes, and causative drugs) in hospitalized elderly surgical patients. The secondary aim was to analyze the severities of the identified DRPs.

## 3. Methods

### 3.1. Setting and Study Population

This retrospective study was conducted at the First Affiliated Hospital of Chongqing Medical University, one of the largest tertiary teaching hospitals of Chongqing, Southwest of China. Inclusion criteria were patients (1) aged ≥65 years, (2) admitted to any of the following six surgical departments (neurology, gynecology, hepatobiliary, vascular, endocrine breast, and orthopedics) from July 1 to December 31 of 2017, and (3) stayed at the surgical department > 1 day and then were directly discharged to home. Exclusion criteria were patients who (1) were transferred to nonsurgery departments and then discharged and (2) had no prescribed medications during the surgical department stay.

### 3.2. The Identification and Classification of DRPs

Electronic prescription orders were reviewed by pharmacists from Monday to Friday, seven hours a day. The prescription review focused on medication indication, contraindication, drug selection, dosage, side effects, and drug-drug interactions. Prescription reviews were provided by pharmacists who had completed clinical pharmacist training programs accredited by the China Ministry of Health, and they had an average of five years' hospital work experience.

DRPs were categorized using the PCNE classification V8.02 [1]. PCNE DRP has five domains: problem type (P), cause (C), intervention (I), acceptance of intervention (A), and outcome (O). In this study, we only investigated the types and causes of DRPs based on study objectives. Two pharmacists each independently categorized DRP types and causes. Discrepancies were discussed between these pharmacists, and a third pharmacist could be consulted to reach a consensus.

The incidence of DRP was defined as the number of elderly patients with at least one DRP divided by the total number of elderly patients in the study multiplied by 100. The incidence was calculated with 95% confidence intervals (CI). The causative medications of DRPs were classified based on the World Health Organization Anatomical Therapeutic Chemical (ATC) classification [8].

### 3.3. Data Collection and DRP Database Construction

Patients' demographics and clinical data, including age, gender, and surgery, were collected from medical records. A DRP database was created using Microsoft Excel to collect prescription data, including DRP problem types (P) and causes (C). The PCNE DRP classification P and C codes were entered into the database by double-checking on entries.

### 3.4. The Assessment of Severities of DRPs

Each DRP was given a severity rating based on the National Coordinating Council for Medication Error Reporting and Prevention (NCC-MERP) classification [9]. NCC-MERP has nine discrete ratings (A-I) that are further combined into four harm categories: (1) circumstances or events that have the capacity to cause errors (category A), (2) medication errors occurred without posing harm to patients or causing potential harm to patients (categories B, C, and D), (3) medication errors caused harm to patients (categories E, F, G, and H), and (4) medication errors resulted in a patient's death (category I). Two pharmacists independently assessed severity ratings, and discrepancies were resolved through discussions with a third pharmacist.

### 3.5. Statistical Analysis

Data were analyzed using SPSS (Version 23.0). Descriptive analysis was performed to summarize patients' demographic and clinical characteristics. Categorical data were expressed as numbers and percentages. Continuous data were expressed as the mean ± standard   deviation.

## 4. Results

### 4.1. Characteristics of Drug-Related Problems

A total of 1,707 elderly patients were admitted to surgery departments throughout the study. Total medication orders reviewed were 53,231 with 1,061 DRPs identified (DRP rate 2.0%). There was an average of 0.6 DRPs per hospitalization, and the vascular surgery department had the most DRPs (42.8%; 454/1,061). A total of 576 elderly patients had at least one DRP. The overall DRP incidence was 33.7% (95% CI, 31.5-36.0). Among patients who had DRPs, 55.4% (319/576) had one DRP, 23.4% (135/576) had two, 11.3% (65/576) had three, 4.3% (25/576) had four, and 5.6% (32/576) had five or more DRPs. In patients with DRPs, the average age was 75.4 years (ranges 65-96), and 53.7% were female.

### 4.2. Drugs Involved in the Occurrence of Drug-Related Problems

The top three drug categories implicated in the DRPs based on the WHO ATC classification (first-level) were “alimentary tract and metabolism A” (31.8%; 337/1,061) followed by “anti-infectives for systemic use J” (23.5%; 249/1,061) and “blood and blood-forming organs B” (22.4%; 238/1,061), respectively. As for the therapeutic subgroups (second-level), “antibacterials for systemic use J01” had the highest DRP percentage (22.1%; 234/1,061) followed by “other alimentary tract and metabolism products A16” (21.5%; 228/1,061) and “peripheral vasodilators C04” (10.3%; 109/1,061), respectively (Table 1).

### 4.3. The Types and Causes of Drug-Related Problems

As shown in Table 2, the primary DRP type was “treatment safety P2” (44.9%; 476/1,061) followed by “treatment effectiveness P1” (31.3%; 332/1,061) and “others P3” (23.8%; 253/1,061). Within the P2 category, “adverse drug event (possibly) occurring P2.1” (44.9%; 476/1,061) occurred the most. The “effect of drug treatment not optimal P1.2” (25.1%; 267/1,061) and “unnecessary drug-treatment P3.2” (19.0%; 202/1,061) were the dominant types in the P2 and P3 categories, respectively.

A total of 1,061 DRP causes were identified. “Drug selection C1” was the major DRP cause followed by “dose selection C3,” each at 43.1% (457/1,061). Within the C1 category, the principal subcategory was “no indication for drug C1.3” (22.3%; 237/1,061). Within the C3 category, “dosage regimen not frequent enough C3.3” (17.7%; 188/1,061) and “drug dose too high C3.2” (16.7%; 177/1,061) were the most common subtypes.

### 4.4. Severities of the Identified Drug-Related Problems

The potential severity ratings of the DRPs (*n* = 1,061) were generally distributed in categories B to H. The percentages from high to low in severity were categories B to D, 75.1% (797/1,061), and categories E to H 24.9% (264/1,061). Within the “drug selection” domain C1, 78.6% (359/457) of the DRPs were classified into severity categories B to D, and 21.4% (98/457) were classified as categories E to H (Table 3).

## 5. Discussions

To the best of our knowledge, this is the first study describing DRPs in elderly surgical patients in a Chinese hospital using the PCNE classification. The presence of DRPs was frequent in this patient population, about one-third of the patients. Almost half of the DRPs occurred in the vascular surgery department. The findings highlight the need for enhanced pharmacy service in this vulnerable patient population.

In the present research, the rate of DRPs was 2.0% of the total prescriptions reviewed, and the mean DRP per patient was 0.6. We could not find similar studies analyzing DRPs in elderly surgical patients for direct comparisons. However, both numbers are higher than what was reported in our previous study (all adult surgical patients, including elderly patients), 1.2% and 0.3 [3]. This shows that elderly patients are more prone to present with DRPs. Pharmacists should target this high-risk population when facing limited pharmacy resources. A study focused on elderly nursing patients in Malaysia reported an average number of DRPs per patient (1.27), higher than our data [10]. The difference may be related to the differences in study design, setting, and the patient population.

The primary causes of DRPs were drug selection and dose selection, almost 90% in this study. Due to age-related pharmacokinetics and pharmacodynamics changes, elderly patients may respond to drug therapy differently compared to general adults. A careful prospective medication review is needed to enhance prescription quality, especially for appropriate drug selection and drug dose. Within the “drug selection” domain, “no indication for a drug” accounted for a higher proportion of all the DRPs, 22.3%. For example, ornithine aspartate injection was given to geriatric patients with gallstones. No indication of drugs may be a unique phenomenon in many developing countries, including China [11]. Ancillary drugs, Chinese patent medicines, antibiotics, and hormones are often overprescribed in China. These drugs may be prescribed with the intent to relieve pain, promote a quick recovery, or profit-driven behavior [12]. Within the “dose selection” domain, “dosage regimen not frequent enough” was the primary cause. For example, cefoxitin sodium twice daily was often prescribed for mild infections, although it should be administered every eight hours due to its short half-life.

Anti-infectives and blood and blood-forming organ medications accounted for almost 50% of the drugs implicated in DRPs based on the ATC classification. Anti-infectives, primarily antibacterials, were commonly prescribed as prophylactic or treatment medications pre- and postsurgeries. Blood substitutes were also frequently used in surgery patients. About one-third of our patients were admitted to the hepatobiliary surgical department, explaining the higher involvement of the alimentary tract and metabolism medications related to the DRP occurrences.

About 75% of the DRPs were rated as severity levels causing no harm or potentially harming patients (categories B to D). Nevertheless, still, 25% of the DRPs might have resulted in harm to patients (categories E to H) requiring intervention or prolonged hospitalization. Because of the retrospective review of the study, the incidence of DRPs associated with actual harms was challenging to obtain.

Our study has the following limitations: (1) this is a single-center study conducted on elderly surgical patients, and the findings may not be extrapolated to other settings; (2) the accuracy in DRP reporting depended on the skills and experiences of individual pharmacists performing this study; (3) the nature of a subgroup analysis of previous data; and (4) only DRP types and causes were investigated. We did not analyze the pharmacists' interventions to resolve the DRPs and the clinical outcomes of patients with the resolution of DRPs.

Despite these limitations, our study reveals a vulnerable patient population that deserves further research. Future prospective, large-scale, and multicenter studies are necessary to evaluate pharmacists' interventions for patients at high risk of potential DRPs. These studies should also assess the clinical, economic, and humanistic outcomes of pharmacy services in elderly surgery patients within the Chinese healthcare system.

## 6. Conclusion

DRPs are common in elderly surgical patients in the hospital setting, and the rate of DRPs is higher than the general population. Pharmacists should provide medication reviews to this vulnerable high-risk population, especially when facing limited pharmacy resources.

## Figures and Tables

**Table 1:** The characteristics of drug-related problems (*n* = 1,061).

	Value
Gender, *n* (%)	
Female	570 (53.7)
Male	491 (46.3)
Age, mean ± SD	75.4 ± 7.0
Number of patients by age group, *n* (%)	
65 to 69 years	246 (23.2)
70 to 74 years	284 (26.8)
75 to 79 years	233 (22.0)
>80 years	298 (28.1)
Admission department, *n* (%)	
Gynecology	33 (3.1)
Hepatobiliary	399 (37.6)
Orthopedics	154 (14.5)
Endocrine breast	8 (0.8)
Neurosurgery	13 (1.2)
Vascular	454 (42.8)
ATC codes of drugs causing DRPs	Number of drugs (%)
A: alimentary tract and metabolism	337 (31.8)
A02 drugs for acid-related disorders	17 (1.6)
A03 drugs for functional gastrointestinal disorders	1 (0.1)
A04 antiemetics and antinauseants	5 (0.5)
A05 bile and liver therapy	41 (3.9)
A10 drugs used in diabetes	7 (0.7)
A12 mineral supplements	38 (3.6)
A16 other alimentary tract and metabolism products	228 (21.5)
B: blood and blood-forming organs	238 (22.4)
B01 antithrombotic agents	30 (2.8)
B02 antihemorrhagics	88 (8.3)
B03 antianemic preparations	43 (4.1)
B05 blood substitutes and perfusion solutions	77 (7.3)
C: cardiovascular system	126 (11.9)
C01 cardiac therapy	1 (0.1)
C02 antihypertensive	2 (0.2)
C04 peripheral vasodilators	109 (10.3)
C05 vasoprotectives	13 (1.2)
C10 lipid-modifying agents	1 (0.1)
D: dermatologicals	1 (0.1)
D04 antipruritics, incl. antihistamines and anesthetics	1 (0.1)
G: genitourinary system and sex hormones	1 (0.1)
G04 urologicals	1 (0.1)
H: systemic hormonal preparations excl. sex hormones and insulins	7 (0.7)
H02 corticosteroids for systemic use	7 (0.7)
J: anti-infectives for systemic use	249 (23.5)
J01 antibacterials for systemic use	234 (22.1)
J04 antimycobacterials	8 (0.8)
J05 antivirals for systemic	7 (0.7)
L: antineoplasic and immunomodulating agents	6 (0.6)
L03 imostimulants	6 (0.6)
M: musculoskeletal system	6 (0.6)
M01 anti-inflammatory and antirheumatic products	2 (0.2)
M05 drugs for treatment of bone diseases	4 (0.4)
N: nervous system	50 (4.7)
N02 analgesics	5 (0.5)
N05 psycholeptics	4 (0.4)
N07 other nervous system drugs	41 (3.9)
R: respiratory system	26 (2.5)
R03 drugs for obstructive airway diseases	26 (2.5)
V: various	14 (1.3)
V03 all other therapeutic products	5 (0.5)
V06 general nutrients	6 (0.6)
V08 contrast media	3 (0.3)

ATC: anatomical therapeutic chemical classification; DRPs: drug-related problems; SD: standard deviation.

**Table 2:** Detected drug-related problems (*n* = 1,061) according to the PCNE DRP classification V8.02.

Primary domain	Code	Detailed classification	*n*	%
(1) Treatment effectiveness There is a (potential) problem with the (lack of) effect of the pharmacotherapy	P1	Total	332	31.3
	P1.1	No effect of drug treatment	40	3.8
	P1.2	Effect of drug treatment not optimal	267	25.1
	P1.3	Untreated symptoms or indication	25	2.4
(2) Treatment safety Patient suffers, or could suffer, from an adverse drug event	P2	Total	476	44.9
	P2.1	Adverse drug event (possibly) occurring	476	44.9
(3) Others	P3	Total	253	23.8
	P3.1	Problem with cost-effectiveness of the treatment	33	3.1
	P3.2	Unnecessary drug treatment	202	19.0
	P3.3	Unclear problem/complaint. Further clarification necessary (please use as escape only)	18	1.7
Total			1061	100.0

PCNE: Pharmaceutical Care Network Europe; DRP: drug-related problem.

**Table 3:** Causes of DRPs (*n* = 1,061) according to the PCNE DRP classification tool and severities of DRPs (*n* = 1,061) based on the NCC-MERP classification.

Primary domain	Code	Causes	*n*	%	Potential severity category B-D (*n*)	%	Potential severity category E-H (*n*)	%
(1) Drug selection	C1	Total	457	43.1	359	78.6	98	21.4
	C1.1	Inappropriate drug according to guidelines/formulary	37	3.5	23	62.2	14	37.8
	C1.2	Inappropriate drug according to guidelines/formulary	38	3.6	28	73.7	10	26.3
	C1.3	No indication for drug	237	22.3	198	83.5	39	16.5
	C1.4	Inappropriate combination of drugs or drugs and herbal medication	40	3.8	34	85.0	6	15.0
	C1.5	Inappropriate duplication of therapeutic group or active ingredient	62	5.8	45	72.6	17	27.4
	C1.6	No drug treatment in spite of existing indication	25	2.4	18	72.0	7	28.0
	C1.7	Too many drugs prescribed for indication	18	1.7	13	72.2	5	27.8
(2) Drug form	C2	Total	86	8.1	77	89.5	9	10.5
	C2.1	Inappropriate drug form (for this patient)	86	8.1	77	89.5	9	10.5
(3) Dose selection	C3	Total	457	43.1	305	66.7	152	33.3
	C3.1	Drug dose too low	15	1.4	10	66.7	5	33.3
	C3.2	Drug dose too high	177	16.7	110	62.1	67	37.9
	C3.3	Dosage regimen not frequent enough	188	17.7	126	67.0	62	33.0
	C3.4	Dosage regimen too frequent	47	4.5	29	61.7	18	38.3
	C3.5	Dose timing instructions wrong, unclear, or missing	30	2.8	30	100.0	0	0.0
(4) Treatment duration	C4	Total	42	4.0	38	90.5	4	9.5
	C4.2	Duration of treatment too long	42	4.0	38	90.5	4	9.5
(8) Other	C8	Total	19	1.7	18	94.7	1	5.3
	C8.2	Other cause	19	1.7	18	94.7	1	5.3
Total			1061	100.0	797	75.1	264	24.9

PCNE: Pharmaceutical Care Network Europe; DRP: drug-related problem.

## Data Availability

The datasets used and/or analyzed during the current study are available from the corresponding authors on reasonable request.
